# Probiotics and Prebiotics Orally Assumed as Disease Modifiers for Stable Mild Atopic Dermatitis: An Italian Real-Life, Multicenter, Retrospective, Observational Study

**DOI:** 10.3390/medicina59122080

**Published:** 2023-11-27

**Authors:** Delia Colombo, Corinna Rigoni, Alessandra Cantù, Antonello Carnevali, Rossella Filippetti, Tiziana Franco, Alessandra Grassi, Camilla Loi, Annamaria Mazzotta, Ivona Patroi, Beatrice Raone, Marco Andrea Tomassini, Angela Amoruso, Marco Pane, Giovanni Damiani

**Affiliations:** 1 Independent Researcher, Private Practice, Via Livigno 6, 20158 Milan, Italy; studio.deliacolombo@gmail.com; 2 Independent Researcher, Private Practice, Corso Monteforte 40, 20122 Milan, Italy; c.rigoni@twtnet.com; 3 Independent Researcher, Private Practice, Via Domodossola 9/A, 20145 Milan, Italy; alessandracantu@yahoo.it; 4 Independent Researcher, Private Practice, Str. Colomba Pecorari 32/a, 06134 Perugia, Italy; acarnevali53@gmail.com; 5 Private Practice, Via Ruspoli 62, 00149 Rome, Italy; cicalafilippetti@yahoo.it; 6 Independent Researcher, Private Practice, Viao Veio, 04100 Latina, Italy; tiziana-franco@libero.it; 7 Independent Researcher, Private Practice, Via Coletti 19, 00191 Rome, Italy; giardinidilussemburgo@gmail.com; 8 Independent Researcher, Private Practice, Via X Settembre 1943 7 and 9, 40011 Anzola dell’Emilia, Italy; camilla.loi30@gmail.com; 9 Independent Researcher, Private Practice, Viale di Villa Massimo 48, 00161 Rome, Italy; a.mazzotta@inwind.it; 10 Independent Researcher, Private Practice, Via del Tritone 102, 00187 Rome, Italy; info@dermatologaivonapatroi.it; 11 Independent Researcher, Private Practice, Via Ruggero Leoncavallo 5, 40137 Bologna, Italy; braone70@gmail.com; 12 Independent Researcher, Private Practice, Via Ponchielli 30, 06073 Corciano, Italy; marcoandreatomassini@gmail.com; 13 Independent Researcher, Probiotical Research srl, Via Mattei 3, 28100 Novara, Italy; a.amoruso@probiotical.com (A.A.); m.pane@probiotical.com (M.P.); 14 Department of Biomedical, Surgical and Dental Sciences University of Milan, 20122 Milan, Italy; 15 Department of Pharmaceutical and Pharmacological Sciences, PhD Degree Program in Pharmacological Sciences, University of Padua, 35122 Padua, Italy

**Keywords:** atopic dermatitis, probiotics, prebiotics, *Bifidobacterium animalis subsp. lactis* BS01, *Lactiplantibacillus plantarum* LP14, *Lacticaseibacillus rhamnosus* LR05, mild atopic dermatitis, prevention

## Abstract

The role of the skin–gut axis in atopic dermatitis (AD) remains a subject of debate, limiting non-pharmacological interventions such as probiotics and prebiotics. To improve understanding of their potential as a monotherapy for stable mild cases, we conducted a real-life, multicenter, retrospective observational study in Italy. We administered three selected bacteria (*Bifidobacterium animalis subsp. lactis* BS01, *Lactiplantibacillus plantarum* LP14, and *Lacticaseibacillus rhamnosus* LR05) orally to patients with mild atopic dermatitis without a placebo control group, following up for 12 weeks. Clinical assessments using the Scoring Atopic Dermatitis (SCORAD), Eczema Area and Severity Index (EASI), and Three-Item Severity (TIS) score were conducted on 144 enrolled patients (average age: 25.1 ± 17.6 years). Notably, both pruritus and AD-related lesions (erythema, edema/papules, excoriation) exhibited significant clinical and statistical improvement (*p* < 0.001) after 12 weeks of exclusive probiotic and prebiotic use. These preliminary results suggest a potential link between the skin–gut microbiome and support the rationale for using specific probiotics and prebiotics in mild AD, even for maintenance, to reduce flares and dysbiosis.

## 1. Introduction

Atopic dermatitis (AD) is a systemic disorder that can manifest as cutaneous (atopic dermatitis), ocular (conjunctivitis), respiratory (allergic asthma, nasal polyposis, and rhinitis), and gastrointestinal (food allergies) symptoms [1]. Atopic dermatitis arises from a complex interplay of genetic and environmental factors. These factors include: defects in the skin barrier function that render the skin more susceptible to irritation from soap and other contact irritants, weather conditions, temperature, and nonspecific triggers; an altered composition of lipids (ceramides) in the stratum corneum; imbalances in protease activity; alterations in the immune system with a bias towards Th2 response and overexpression of cytokines IL-4, IL-5, and IL-31; and elevated levels of CD4 and CD25 lymphocytes with reduced CD8 levels [1].

Several molecular markers expressed in the skin have been implicated in the pathogenesis of eczema, including filaggrin, aquaporin-3, and interleukin-31. Filaggrin, a filament-aggregating protein, is a key protein that plays a crucial role in the formation of the cornified cell envelope, which is essential for an effective skin barrier. Compared to normal skin, filaggrin expression is significantly reduced in acute eczema. Aquaporin-3 (AQP3) is a water and glycerol transporter expressed in the plasma membranes of keratinocytes in the basal layer of the epidermis in normal skin. Increased expression and altered cellular distribution of AQP3 have been found in eczema, which may contribute to water loss. Interleukin-31 (IL-31), predominantly produced by Th2 cells, is a potent pruritogenic cytokine. Recent clinical studies have revealed that administration of an IL-31 receptor antibody significantly alleviates itchiness in patients with eczema. In addition to these factors, new research has indicated the involvement of other genetic variants, epigenetic modifications, environmental triggers such as allergens, microbial dysbiosis, and the role of neuroimmune interactions in the pathogenesis of atopic dermatitis. Understanding the etiopathogenesis of AD is crucial for the development of targeted therapies and interventions for this chronic and debilitating condition [1].

In 1989, the hygiene hypothesis was introduced, suggesting that the increase in allergic diseases observed in recent decades could be attributed to a lack of microbial exposure, particularly during early childhood, associated with improved hygiene practices. Behavioral changes for hygiene and public health have led to an unprecedented era of cleanliness and the near eradication of previously common pathogens. These improvements have coincided with the rise of autoimmune diseases and other immune-related disorders.

According to the hygiene hypothesis, a priming of the immune system through essential immunomodulatory exposures helps stimulate regulatory mechanisms that protect against infectious diseases and allergies. Even the definition of allergy itself has evolved over the years and is currently considered a broad term for an immune defect resulting in a lack of tolerance to usually harmless antigens. Tolerance is antigen-specific, and the loss of tolerance in allergies appears to be related to the timing, speed, and context of environmental exposures in early life, including the bacterial colonization of the infant gut [2].

Despite the systemic nature of the disorder, clinical approaches have historically been compartmentalized due to a lack of comprehensive scientific evidence [2]. However, recent insights into the role of the microbiome in maintaining tissue health have shifted the perspective on the hyper-hygienist theory, opening up possibilities for the use of prebiotics, probiotics, and postbiotics [3]. Studies indicate interdependence between microbiomes in different tissues (i.e., skin and gut), suggesting the presence of modulatory axes capable of influencing inflammation [4,5].

Strategies to address perturbed cutaneous microbiomes, such as those capable of triggering AD flares, remain crucial, especially for probiotics involving live bacteria [6,7]. Intestinal colonization by bacteria with pro-Th1 or pro-Th2 properties may play a role in the development of responses against intracellular pathogens and in the prevention of diseases characterized by Th1/Th2 imbalances, such as allergic disorders and autoimmune diseases [8,9]. In light of these dynamics, this study aimed to evaluate the potential effects of specific bacteria (*Bifidobacterium animalis subsp. lactis* BS01, *Lactiplantibacillus plantarum* LP14, *Lacticaseibacillus rhamnosus* LR05) in addressing gut dysbiosis associated with mild atopic dermatitis [10].

## 2. Methods

### 2.1. Ethical Considerations

The research protocol adhered to the ethical principles outlined in the Declaration of Helsinki and its subsequent amendments, governing medical research involving human subjects. All participants provided informed consent prior to their involvement in the study. The study was classified as a post hoc analysis of a previously evaluated and endorsed study by the Ethical Committee of San Raphael Hospital (178/INT/2021) on 10 November 2021.

### 2.2. Study Design

This study represents a real-life, multicenter, retrospective observational investigation designed to assess the effectiveness and tolerability of a commercially available pre- and postbiotic supplement (Atopicina^®^, Funziona s.r.l, Milan, Italy).

The experimental group received 90 sachets of the product, with each sachet intended for daily consumption. These sachets contained a concentration exceeding 2.5 × 10^9^ AFU (active fluorescent units) of three patented probiotic species: *Bifidobacterium animalis subsp. lactis BS01* (LMG P-21384), *Lacticaseibacillus rhamnosus LR05* (DSM 19739), and *Lactiplantibacillus plantarum LP14* (DSM 33401). The three strains were chosen due to their synergistic effect on IL-10 production, an anti-inflammatory cytokine that plays a pivotal role in modeling the gut and skin microbiome.

These probiotics were suspended in 2.6 g of a freeze-dried powder mixture containing FOS (comprising approximately 96% of the total weight) and vitamin B2 (Probiotical S.p.A., Novara, Italy). Participants were directed to dissolve the powder only in water and consume it during breakfast to avoid circadian rhythm confounders. The probiotic sachets underwent analysis by Biolab Research S.r.l. (Novara, Italy) using flow cytometry (ISO 19344:2015 IDF 232:2015) to verify the specified target cell count of ≥2.5 × 10 ^9^ AFU. Continuous monitoring of product stability ensured maintenance of minimum cell counts. 

The study spanned from December 2021 to October 2022, encompassing a 6-month follow-up duration. In-person clinical evaluations were conducted at three time points within the 4-month follow-up period: T0 (baseline), T1 (after 4 weeks), and T2 (after 12 weeks). For the purpose of enhancing sample diversity and the applicability of outcomes, exclusively dermatological private practices in Italy were enlisted.

No placebo control group was planned due to ethical concerns.

### 2.3. Inclusion and Exclusion Criteria

The study incorporated patients who fulfilled the following criteria: (a) age exceeding 3 years, (b) mild atopic dermatitis status (as indicated by Scoring Atopic Dermatitis (SCORAD) scores < 20 or Eczema Area and Severity Index (EASI) scores < 7) [11], (c) stable AD (Delta SCORAD between two consecutive measures < 15%), and (d) provision of informed consent (in line with Italian law, for pediatric patients or patients legally unable to understand, the signatures of the parents or the legal tutors were collected). On the contrary, individuals with (a) chronic inflammatory, infectious, or oncological disorders; (b) multiple chemical sensitivity (MCS) [12]; (c) a documented allergy to the supplement’s components; (d) utilization of topical steroids, or other supplements or antibiotics within the preceding two weeks; or (e) refusal to grant informed consent were excluded. Importantly, the study involved a diverse range of patients, including those of Caucasian, African, and Asian descent, to yield robust and comprehensive data.

Since emollients are the basis of proper skincare in AD patients, the dermatologists also suggested that the enrolled patients apply only a ceramides-based emollient (the emollient used was Ceramol 311 Cremabase 400 mL) daily in accordance with their skin type (oily, dry, normal, combination, and sensitive skin) [13,14,15,16]. The emollient prescribed was marketed and non-galenic. In contrast, moisturizers were not allowed. Furthermore, SPF 100+ sunscreen (infrared covered) was introduced into the daily care of all participants and re-applied every 6 h to prevent photoaging and photo-induced inflammation in erythematous areas [17,18,19,20,21,22]. The sunscreen was applied in photo-exposed areas during the whole duration of the study, and during the summer all participants wore UVB protective clothes [23,24,25].

### 2.4. Clinical Assessment

All patients included in the study were individually evaluated in person by two board-certified dermatologists, both native Italian speakers. Detailed data encompassing demographics, medical histories, and clinical information were meticulously collected.

In addition to SCORAD and EASI assessment, the severity of symptoms was appraised using the Three-Item Severity (TIS) score, which classifies severity as mild (<3 points), moderate (3–5 points), or severe (6–9 points) [26]. The evaluation was further refined by quantifying erythema and excoriations on a scale from 0 (absent) to 4 (severe). TIS was preferred over SCORAD and EASI due to its greater precision in detecting clinical changes within this specific subset of mild atopic dermatitis patients. The extent of pruritus was evaluated and tracked using the PRURISCORE analogic scale, a six-point scale visually representing itch intensity with an explanatory label [27]. Labels used to explain the PRURISCORE were as follows:

- No itching;
- Very mild itching: scratching can be avoided;
- Mild itching: scratching is occasional;
- Moderate itching: scratching is constant and rest is disturbed;
- Severe itching: scratching is intense and provokes skin marks and, at the same time, rest is very disturbed;
- Intolerable itching: scratching is violent and provokes excoriations and, at the same time, rest is impossible.

### 2.5. Statistical Analysis

A per-protocol statistical analysis was undertaken employing nonparametric statistical methods. Variables including the PRURISCORE, erythema, papules, edema, and excoriation were subjected to analysis using the Friedman test, followed by the Dunnett *t*-test.

The presentation of results utilized the mean ± SD. Observed disparities were deemed statistically significant when *p*-values were less than 0.05.

## 3. Results

In the current investigation, a total of 144 patients were enrolled, demonstrating a slight female predominance (*N* = 77, 53.5%). The average age of the enrolled participants was 25.1 ± 17.6 years (Table 1).

Notably, all employed severity scores (erythema, edema/papules, excoriation, TIS, and PRURISCORE) exhibited a substantial overall and even intra-individual reduction over the course of the study, underscoring the robust and favorable impact of pre- and probiotic supplementation (Table 2).

Specifically, the initial PRURISCORE of 9 at T0 exhibited a decline from 3.04 ± 0.10 to 2.01 ± 0.95 at T1 and further to 0.99 ± 0.93 at T2 (*p* < 0.001). This downward trend was mirrored when evaluating erythema, edema-papules, and excoriation, all of which displayed statistically significant reductions (*p* < 0.001).

Consistently, these trends were upheld by the TIS score (*p* < 0.001). At baseline (T0), the TIS score stood at 5.45 ± 2.00, then diminished to 3.30 ± 1.82 after 4 weeks (T1) (*p* < 0.05), and ultimately reached 1.42 ± 1.41 after 12 weeks (T2) (*p* < 0.05). Likewise, the SCORAD (T0: 11.3 ± 0.9, T1: 7.1 ± 1.3, T2: 2.8 ± 0.6, *p* < 0.001) and EASI (T0: 3.7 ± 1.2, T1: 2.4 ± 0.8, T2: 1.1 ± 0.3, *p* < 0.001) differences between the different timelines were statistically significant.

**Figure 1:**
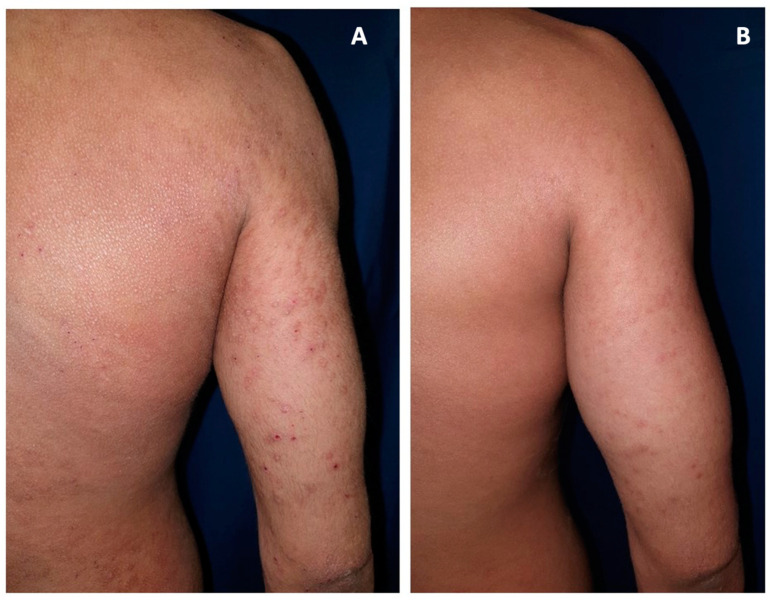
A 32-year-old patient at the baseline (T0, (**A**)) and after 12 weeks (T2, (**B**)) of using Atopicina^®^, which dramatically improved itch, erythema, and papules. In T0 several excoriated erythematous papules appear in the extensor area of the arm, that resolved leaving mild hyperpigmented macules in T1 after Atopicina^®^ treatment.

Patients displayed a cutaneous improvement in lesion severity (Figure 1A,B).

Significantly, it is noteworthy that within our patient cohort, neither dropouts nor adverse effects were identified.

## 4. Discussion

Mild atopic dermatitis benefits from oral pre- and probiotics that can counteract skin barrier defects, thus preventing clinical signs of AD, such as erythema. Recently, dysbiosis in the intestinal microbiome has been recognized as a potential initiator or modulator of cutaneous barrier issues, suggesting the potential for oral agents to modulate atopic dermatitis [28]. While previous topical strategies involving pre-, pro-, and postbiotics have shown promising results in vitro, they have fallen short as effective real-life disease modifiers [29].

The gut microbiome can be seen as the primary filter mediating interactions with environmental factors, including dietary elements. Additionally, both the gut and skin microbiomes are influenced during natural childbirth and play a role in forming cutaneous/mucosal barriers in children [30]. Disruption of this delicate process could lead to the development of AD and a compromised resident immune system. Simultaneously, the microbiome is a living and dynamic entity, serving as continuous training for the local immune system, evolving alongside the environment [31]. Bacterial catabolites have vasoactive properties and can trigger distant clinical signs, such as erythema in erythematotelangiectatic rosacea [32] or in small intestinal bacterial overgrowth (SIBO) for psoriasis [33].

Furthermore, bacteria within the gut interact with other microbes to create biofilm tridimensional structures that influence antigen exposure to the local immune system [34]. However, excessive exposure to bacteria that evade the immune system may trigger cross-reactivity and subsequent autoimmunity [35]. Given these dynamics, addressing gut dysbiosis in AD patients becomes crucial. This intervention could synergize with topicals and even targeted therapies that address specific pathological aspects, such as barrier dysfunction or Th-2 inflammation [36,37].

In line with existing literature, AD patients often experience gut dysbiosis, leading to instability in the microbiome and creating an environment conducive to the introduction of orally ingested bacteria, as seen in this study. This phenomenon potentially explains the clinical outcomes observed in our study after oral supplementation with *Lactiplantibacillus plantarum* LP14 (DSM 33401), *Bifidobacterium animalis subsp. lactis* BS01 (LMG P-21384), and *Lacticaseibacillus rhamnosus* LR05 (DSM 19739).

While this study’s innovative design is noteworthy, it does have limitations, such as the absence of baseline and post-intervention monitoring of the cutaneous microbiome and a placebo control group. Nevertheless, this clinical study establishes a strong link between the use of the evaluated supplement and the absence of AD flares in the enrolled patients. Future studies should delve deeper into the pathway modulation of Atopicina^®^, which is potentially capable of preventing AD flares.

The decision to conduct this study in a more diverse setting, involving dermatologists from private clinics instead of university clinics, was deliberate. To facilitate practicality in an outpatient setting for mild AD, we chose to use the Three-Item Severity (TIS) scale, which is more user-friendly than the SCORAD index. Similarly, the adoption of the PRURISCORE scale was motivated by its immediacy and ease of use during patient interactions [38].

It is important to note that this study was conducted without any compensation for the participating dermatologists. Despite not performing circulating and local cytokine assays due to the pilot design (real-life centered), we observed significant improvements in the symptoms and severity of atopic dermatitis. These findings support the notion that targeting the gut–skin axis through probiotic and prebiotic interventions may hold great potential in managing atopic dermatitis. Furthermore, our study highlights the importance of specific probiotics in modulating the Th1–Th2 imbalance observed in atopic individuals. By restoring immune homeostasis, these probiotics may contribute to the amelioration of atopic dermatitis symptoms and potentially address the underlying immune dysregulation in other manifestations of atopy. Considering the interconnected nature of atopic conditions, it would be of great interest to conduct further clinical studies on other manifestations of atopy, such as allergic rhinitis and asthma. Exploring the effects of this symbiotic formulation on these conditions could provide valuable insights into the broader applications and therapeutic potential of targeting the gut microbiome in managing atopic diseases comprehensively. It is important to note that our study prioritized the safety of the symbiotic product, and no significant adverse effects were observed. This reinforces the notion that the symbiotic formulation used in this study is well-tolerated and supports its potential as a safe therapeutic option for individuals with atopic dermatitis.

In conclusion, our findings highlight the promising efficacy of the specific symbiotic formulation in improving symptoms and modulating the gut microbiome in atopic dermatitis. The potential for extending these benefits to other atopic conditions warrants further investigation and may open new avenues for personalized approaches in managing the complex spectrum of atopic diseases.

## Figures and Tables

**Table 1:** Clinical and demographic characteristics of the enrolled patients.

Characteristics of the Enrolled Patients	
Gender:	
Male (*N*; %)	66; 45.8
Female (*N*; %)	78; 55.2
Age (average ± SD)	25.1 ± 17.6
Family History (*N*; %)	29; 20.1
Asthma (*N*; %)	1; 0.7

SD: Standard deviation.

**Table 2:** Clinical data of AD patients.

T0 (Baseline)	T1 (4 Weeks)	T2 (12 Weeks)	X^2^_r_	*p*
Erythema				
2.00 ± 0.741	1.27 ± 0.719	0.59 ± 0.641	194.6	<0.001
Edema/papules				
1.64 ± 0.902	0.93 ± 0.774	0.39 ± 0.589	170.2	<0.001
Excoriation				
1.86 ± 0.875	1.08 ± 0.731	0.45 ± 0.609	190.2	<0.001
TIS score				
5.49 ± 1.98	3.28 ± 1.88	1.43 ± 1.47	239.1	<0.001
PRURISCORE				
3.04 ± 0.993	2.00 ± 0.998	0.96 ± 0.919	148.1	<0.001

TIS: Three-Item Severity.

## Data Availability

The data presented in this study are available on request from the corresponding author. The data are not publicly available due to privacy restrictions.
